# A Randomized Trial of Instructor-Led Training Versus Video Lesson in Training Health Care Providers in Proper Donning and Doffing of Personal Protective Equipment

**DOI:** 10.1017/dmp.2020.56

**Published:** 2020-03-30

**Authors:** Liva Christensen, Charlotte Schang Rasmussen, Thomas Benfield, Jeffrey Michael Franc

**Affiliations:** Department of Infectious Diseases, Amager and Hvidovre Hospital, University of Copenhagen, Copenhagen, Denmark; Nykøbing Falster Hospital, Department of Anaesthesiology, Denmark; Faculty of Health and Medical Sciences, University of Copenhagen, Copenhagen, Denmark; University of Alberta, Edmonton, AB; Università del Piemonte Orientale, Novara, Italy

**Keywords:** personal protective equipment, coronavirus, video training, randomized trial

## Abstract

**Objective:**

This study compared live instructor-led training with video-based instruction in personal protective equipment (PPE) donning and doffing. It assessed the difference in performance between (1) attending 1 instructor-led training session in donning and doffing PPE at 1 month prior to assessment, and (2) watching training videos for 1 month.

**Methods:**

This randomized controlled trial pilot study divided 21 medical students and junior doctors into 2 groups. Control group participants attended 1 instructor-led training session. Video group participants watched training videos demonstrating the same procedures, which they could freely watch again at home. After 1 month, a doctor performed a blind evaluation of performance using checklists.

**Results:**

Nineteen participants were assessed after 1 month. The mean donning score was 84.8/100 for the instructor-led group and 88/100 for the video group; mean effect size was 3.2 (95% CI: -7.5 to 9.5). The mean doffing score was 79.1/100 for the instructor-led group and 73.9/100 for the video group; mean effect size was 5.2 (95% CI: -7.6 to 18).

**Conclusion:**

Our study found no significant difference in donning and doffing scores between instructor-led and video lessons. Video training could be a fast and resource-efficient method of training in PPE donning and doffing in responding to the COVID-19 pandemic.

## INTRODUCTION

When working with infectious diseases with high consequences, such as Ebola and COVID-19, the simple act of donning (putting on) and doffing (removing) personal protective equipment (PPE) becomes a lifesaving procedure not only for the medical staff, but also for the thousands of people who depend on them.

In the current COVID-19 pandemic of 2020, with an urgent need to train large numbers of staff in how to don and doff PPE, the use of traditional face-to-face training with an instructor poses a number of challenges. First, it is time-consuming for both trainees and instructors. Second, gathering people together to be trained in person increases the risk of exposing staff to infection.^1^ Third, the rapid growth in numbers of cases of infection and disruptions in the global supply chain of PPE presents a real risk of shortages of this equipment.^2^ Therefore, alternative training methods should be considered.

This study compared live instructor-led training with video-based instruction in PPE donning and doffing. It assessed whether there was a performance difference between a control group that was assessed 1 month after receiving instructor-led training in PPE donning and doffing, and a study group that had access to training videos providing similar instruction over the month prior to assessment.

## ETHICAL APPROVAL

The Ethical Committee on Health Research Ethics of the Capital Region of Denmark reviewed the research protocol and waived the requirement for informed consent because the study subjects were healthy volunteers and the study did not involve an intervention.

## METHODS

This was a randomized controlled trial pilot study of video versus live instructor-led training.

### Participant Population

The study was conducted in Denmark. Volunteers responded to poster advertisements and brochures posted at the Faculty of Health and Medical Sciences, University of Copenhagen, at Hvidovre Hospital and Slagelse Hospital. The posters were also included in a magazine for medical students (MOK; Medical Organizations Communications Agency) and posted online in several medical student and medical Facebook groups.

Twenty-one participants took part in the pilot study. Inclusion criteria were no previous training in donning high-isolation PPE and 3- to 6-year medical students and junior doctors (1-year doctors before receiving their independent license). Exclusion criterion was previous experience in donning high-isolation PPE (PPE created for diseases with high transmission risk).

### Randomization

Randomization was done immediately upon arrival at the training center. Participants who were enrolled into this study were given a sealed envelope containing a unique number. This number was used in data recording throughout the study. The participants were randomly divided into 2 groups by rolling a dice. Those who rolled 1–3 were included in the group with an instructor (control group). Those who rolled 4–6 were included in the group to be trained by watching videos (study group).

Training was given by a nurse, and the evaluation was performed by a doctor. Both had worked for 3 weeks at the Port Loko Ebola treatment center in Sierra Leone in 2015 and had attended an intensive 5-day, pre-departure training in the use of PPE prior to their departure, held in England and organized by the Register of Engineers for Disaster Relief. The instructor had participated in adjusting the checklists used in the evaluation. The evaluator had seen the video made by the instructor. In this way, it was ensured that the evaluator was evaluating according to the same criteria that the instructor had used in the training. To ensure that this was a blind study, the evaluator who evaluated the volunteers did not know which students had been trained by the video method (study group) and which had been trained by the instructor (control group).

### Design

Control group participants each attended one 2- to 3-hour training session during which a demonstration by the instructor of how to don and doff PPE correctly was followed by observation of each of them donning and doffing PPE. The training with the instructor was conducted in groups of 1–4 people and participants donned and doffed PPE several times and received feedback. Before leaving, each participant had to perform sufficient donning and doffing as determined by the instructor, and they needed to confirm that they felt confident with the procedure.

The study-group participants watched a pair of videos about how to don and doff the PPE (donning 4:42 minutes and doffing 6:11 minutes). They were required to watch the video immediately after randomization to ensure that they had seen the video at least once. This initial video training session was assisted by an Information Technology specialist who had the role of ensuring that everyone in the video group was able to access the videos from home. The free access allowed them to watch the videos as many times as they wished, and they were asked to record when (and thus the number of times) they watched the videos.

The videos were made by the same nurse using the same technique and the same equipment that was used in the live training. Both the training and the videos were in Danish. See the following websites:

Donning video: https://www.youtube.com/watch?v=laeXzmkX8L0
Doffing video: https://www.youtube.com/watch?v=AQ-J1HvnquQ

After 1 month, both groups were evaluated using the pair of validated checklists for donning and doffing, “2014 Donning and Doffing PPE Competency Validation Checklist,” by the Association for Professionals in Infection Control and Epidemiology.^3^ The checklists were updated to exactly match the specifics of the PPE used (Table 1, Table 2). The equipment was functionally the same as that which the PPE nurse (instructor) and doctor (evaluator) had used in Sierra Leone.

**TABLE 1:** Updated Donning Checklist

	Participation Number
	Time of Performance (min)
	Levels of Performance (L of P)
	0 Not applicable
	1 Needs assistance (little or no experience)
	2 Minimal assistance (some experience)
	3 Perform independently (competent, experienced)
	4 Resource/instructor (competent/able to teach)
	**Updated Donning PPE Competency Checklist**
1	Performs donning in a clean area
2	Performs hand hygiene
3	Ties hair up and back from face and removes jewelry (to avoid contamination and damaging PPE)
4	Ensures that equipment is not damaged and dons gown (*Note:* All TIES should be properly secured with a SIMPLE BOW.)
5	Dons boots (pulls the outside of the gown over the boots so nothing can fall inside the boots)
6	Applies standard gloves (puts the elastic from gown over the finger to avoid sleeves rolling upward)
7	Finishes donning gown (closes the gown to the top and ensures the zipper is pointing downward; asks buddy to help with rolling down the hood; ensures that all fit well and cover the intended areas)
8	Applies N-95 respirator (seals respirator to the face, ensuring straps are not crossed and are properly located at the crown of the head and base of the neck)
9	Performs a fit check of the respirator
10	Dons surgical hood (assists with holding the hood while buddy closes the surgical hood on the back of the head, binds the 2 long ones in a simple bow on the stomach)
11	Dons apron (asks buddy to close the apron with a simple bow)
12	Applies long cuff gloves over the standard gloves (asks for help from buddy if needed to pull the gloves up to the elbow; puts on elastics to avoid gloves rolling down)
13	Applies goggles (asks buddy to check that there is no skin exposed)
14	Appropriately works with a donning partner
15	Uses donning partner for assistance and to obtain any supplies

**TABLE 2:** Updated Doffing Checklist

	**Participant Number**
	Time
	Levels of Performance (L of P)
	0 Not applicable
	1 Needs assistance (little or no experience)
	2 Minimal assistance (some experience)
	3 Perform independently (competent, experienced)
	4 Resource/instructor (competent/able to teach)
	**Updated Doffing PPE Competency Checklist**
1	Uses gentle, slow technique to remove gown (principle: “clean on clean, dirty on dirty”)
2	Steps into chloride boot wash
3	Steps out of patient room into decontamination room, stays in “URENT” zone with trash receptacle at door
4	Spraying the gloves
5	Body being sprayed
6	Washes hands
7	Removes the long cuff outer gloves using glove-in-glove technique and discards in trash
8	Removes apron by pulling 1 of the ribbons on the back to untie the simple bow, taking in the top corner (not touching the front of the apron) and moving it carefully over the head
9	Discards apron in the bucket with chloride
10	Washes hands
11	Closes eyes and removes the goggles, pulling it forward so it is free from the face, gently pulling it off
12	Discards goggles in the chlorine bucket
13	Washes hands
14	Unties the ribbons on the hood; closes eyes and removes the hood by pulling it forward
15	Throws the hood in the trash
16	Washes hands
17	Opens coverall correctly: (1 + 2 + 3 + 4)
	(1) Opens the gown (without touching the face, therefore start opening from middle)
	(2) Washes hands
	(3) Finds the zipper from middle (not from the top part)
	(4) Opens the zipper
18	Washes hands
19	Removes coverall correctly (principle: “clean on clean, dirty on dirty”); doesn´t pull hands all the way out of sleeves; uses the inside clean part of the sleeves to bring the suit down around the boots; doesn´t touch boots on the dirty and outer side of the suit (1 + 2 + 3 + 4)
	(1) “Clean on clean, dirty on dirty”
	(2) Doesn´t pull hands all the way out of sleeves
	(3) Uses the inside clean part of the sleeves to bring the suit down around the boots
	(4) Doesn´t touch boots on the dirty and outer side of the suit
20	Leaves the suit on the floor for spraying
21	Carefully picks up suit, keeping it away from body and discards it in the trash
22	Washes hands
23	Removes respirator, pulling it forward away from face
24	Discards respirator in the trash
25	Washes hands
26	Removes the inner gloves (principle: “clean on clean, dirty on dirty”)
27	Spraying of boots (both sides)
28	Lifts the foot next to clean area, lets it be sprayed then steps with a clean foot into “REN” zone and lifts the other foot to be sprayed and then also steps with a clean foot into zone
29	Washes hands 0.05% chloride
30	Steps in the other chlorine boot wash

**FIGURE 1:**
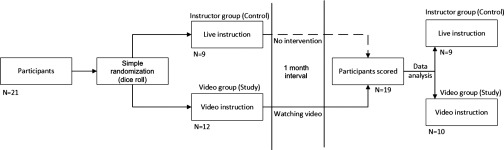
Statistical Methods, Tests, Software.

Participants were informed that they should not tell the evaluator which method they had been trained with. After the evaluation, on leaving the assessment room, video group participants submitted the calendars on which they had recorded when they had viewed the videos on donning and doffing PPE during the past 1 month, together with their unique number (Figure 1).

For continuous values, results are presented as mean with 95% confidence intervals. The differences in scores between the video and instructor groups were assessed using the 2-sample t-test and presented as mean effect size with a 95% confidence interval.

A data analysis was performed using R (R Foundation for Statistical Computing, Vienna, Austria).

## MATERIAL

### Personal Protective Equipment

- Coveralls – Lakeland Europe ChemMax 1 special coverall with hood and loops (elastic)
- Hoods – Microgard hoods with surgical masks WH25/W/00/517/00 (special design, as used in the Ebola treatment center in Sierra Leone)
- Goggles – Progressive safety indirect vent goggles
- Respirators – Seton JSP 111 FFP1 disposable dust mask
- Aprons – Seton chemical resistant apron with ties 48” long x 36” wide
- Inner gloves – Abena nitrile medical examination gloves
- Outer gloves – Gleco medical sterile gynecological gloves, 480 mm long, 0.20 mm thick

## RESULTS

**FIGURE 2:**
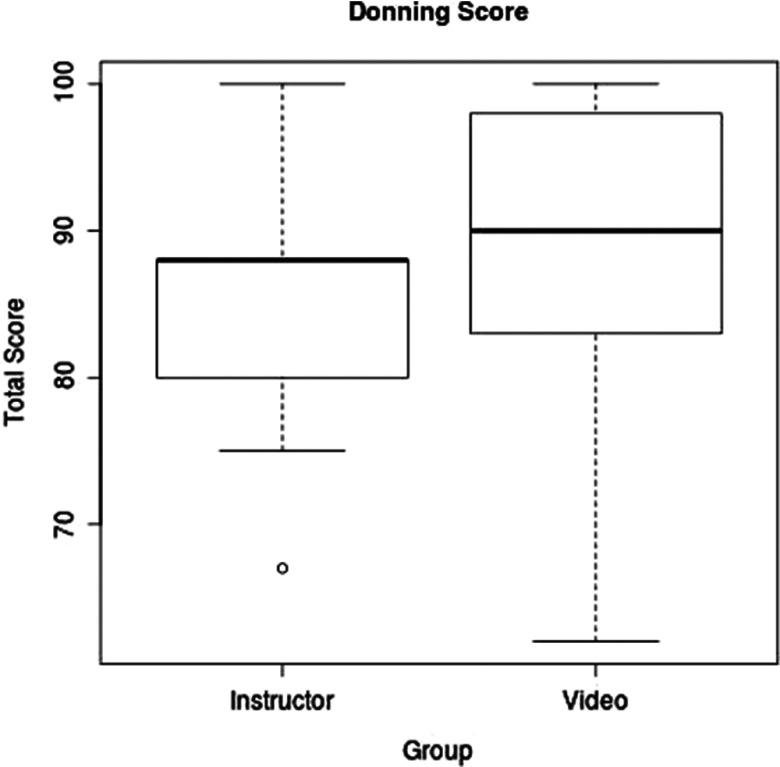
Donning Score by Training Method.

Twenty-one participants received instruction, with 9 in the control (instructor) group and 12 in the study (video) group. Nineteen returned to be evaluated 1 month later: 9 from the control group and 10 from the study group. In donning, the scores in the instructor group ranged from 67% to 100%, and the scores in the video group ranged from 62% to 100%. The overall mean donning score was 86.5/100; the mean score was 84.8 for the instructor group and 88.0 for the video group. There was no statistically significant difference in the donning score between the instructor and video groups (95% confidence interval for the effect: -7.7 to 9.5; *P*-value: 0.54) (Figure 2).

**FIGURE 3:**
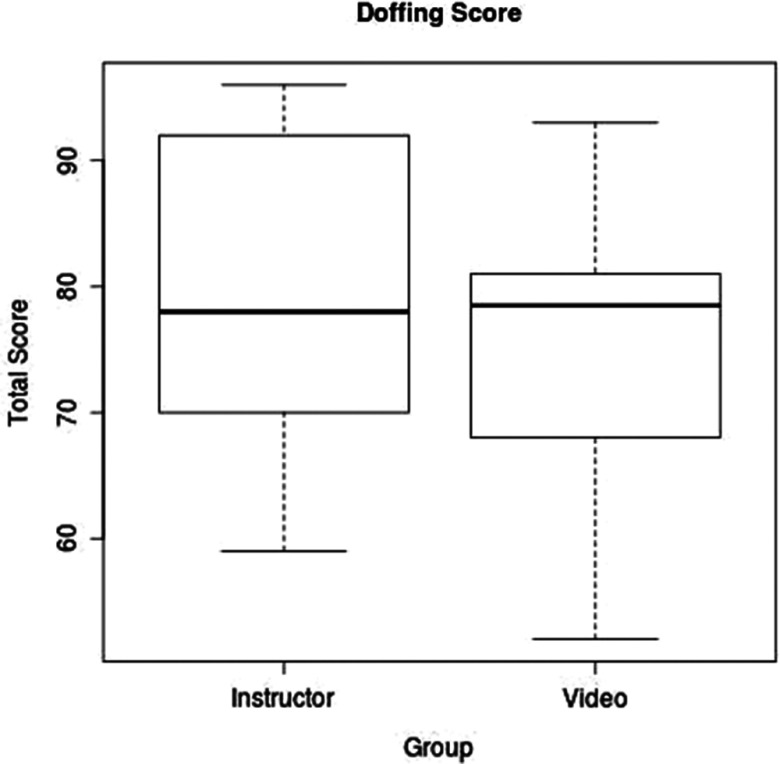
Doffing Score by Training Method.

In doffing, the scores in the instructor group ranged from 59% to 96%, and the scores in the video group ranged from 51% to 93%. The overall mean doffing score was 76.4/100; the mean score for the instructor group was 79.1, and it was 73.9 for the video group. There was no significant difference in the doffing score between the video group and the instructor group (95% confidence interval for effect: -7.6 to 18.0; *P*-value: 0.54) (Figure 3).

The average numbers of times the videos were watched were 4.9 for the donning video and 5.2 for the doffing video. The videos were most watched on the first and last days (Table 3).

**TABLE 3:**
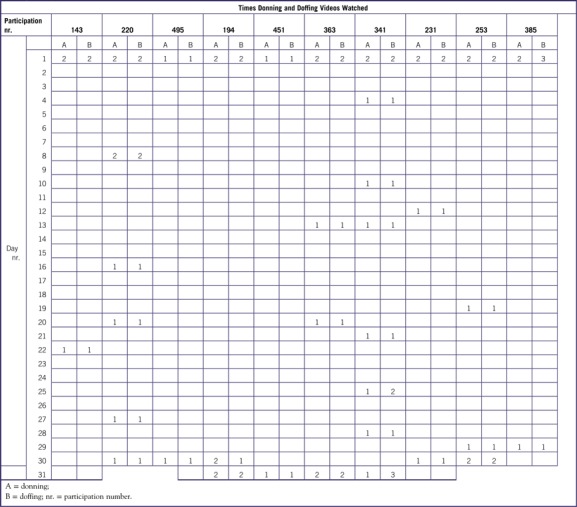
Record of Video Viewing

The average amounts of time spent watching the videos during 1 month were 23 minutes for the donning video and 32 minutes for the doffing video, yielding an average total video training time of 55 minutes. For the instructor (control) group, the training sessions with 1–4 participants took 120–180 minutes.

## DISCUSSION

The participants who received video training were on average as competent as those who received instructor-led training in person. The video training took only around one-third of the time taken for a training session with an instructor. Therefore, in responding to the COVID-19 pandemic, video training might be a resource-efficient way of reaching all relevant personnel without requiring face-to-face training.

Although video group participants could watch the videos as many times as they wished and whenever they wanted, it was observed that most of them had watched the video on the assessment day, effectively receiving “just-in-time” training. This may have helped their performance and might explain why some participants from the video group performed even better than those who had received training with the instructor 1 month previously.

Just-in-time training, whereby information is provided at the time it is needed, has been proven to be effective in teaching other procedures. Jones et al. compared differences in just-in-time training between video presentation and small group demonstrations for fit testing N-95 respirator face masks. Fit testing of N-95 is also a part of PPE donning. There were no differences between the groups when assessment was done immediately.^4^ However, donning and doffing PPE is much more complicated because it consists of procedures that not only need to be performed correctly, but also in the right sequence, and this is of very high importance. Successful performance by video group participants may have been aided by other factors, such as repetition of the material, not only the possibility to study immediately prior to assessment.

Nevertheless, the West Africa Ebola outbreak (2014–2016) showed how training material can be developed quickly during an emergency response and made available for personnel being deployed to the response,^5^ and this study suggests that the rapid development of video materials to provide training in donning and doffing of PPE is worthy of consideration in the case of the COVID-19 outbreak.

### Limitations

To keep the videos down to a reasonable length (donning 4:42 minutes and doffing 6:11 minutes), they needed to be edited. In this process, care was taken not to miss any important aspects. However, feedback from participants revealed that some parts of the videos had been hard to see.

During the group video training on Day 1, participants complained that it was hard to see how to take off the gloves following the “clean on clean, dirty on dirty principle” (see doffing video at 4:56 minutes). Two additional videos were immediately added and all video group participants were given access to them. They gave the same demonstration of how to take off the gloves, but slower and without editing. See the following websites:

Additional video of long glove removal: https://www.youtube.com/watch?v=o5TCt7QXPQo
Additional video of short glove removal: https://www.youtube.com/watch?v=SX9KxvMuI2k

The video was revised in a rapid time frame to correct a poorly visible action, and, although this invites the criticism that the video group received feedback as an answer to a question in a similar manner to personal feedback during live instructor-led training, we consider that this revision did not impact the results of the study.

In the last assessment session, a participant who had been trained by video had watched the doffing video 6 times without noticing that the third ribbon on the neck is untied and therefore received minus 1 point for not untying the ribbon in the assessment. The video was reviewed and it was concluded that the untying of the third ribbon was not properly shown (see doffing video at 2:15 minutes).

Although these 2 events did not significantly impact the results of the study, they highlight the importance of testing videos before use in real training, inviting feedback, and making revisions when errors are found or when new information arises.

Another limitation was that the sample size was small and therefore the confidence intervals for the effect are large. A larger study might show a statistically significant difference between groups.

## CONCLUSION

The study suggests that the PPE donning and doffing competencies of 2 groups were similar where 1 group received live training from an instructor for 1 month before being assessed and the other group watched videos of similar instruction multiple times during the month prior to assessment. The data demonstrate that the video method is time- and resource-effective when training many participants. In responding to the current COVID-19 pandemic in 2020, video training in donning and doffing PPE could provide a means of training large numbers of personnel, while minimizing the amount of time and PPE used in training and ensuring social distancing. This study also highlights the need for training videos to be tested to ensure completeness, accuracy, and clarity of actions.

Although the focus of this study was not just-in-time training, the observation that most of the video group participants watched the training videos on assessment day suggests that this may have been a beneficial factor in their performance. The effect of different video training schedules and strategies on performance in donning and doffing PPE would be worthy of further study.

## Abbreviation

PPE: personal protective equipment
